# Electronic Cigarettes in Thailand: Behaviour, Rationale, Satisfaction, and Sex Differences †

**DOI:** 10.3390/ijerph19148229

**Published:** 2022-07-06

**Authors:** Tamonwan Chankaew, Peeraporn Baiya, Dujrudee Chinwong, Voratima Yoodee, Surarong Chinwong

**Affiliations:** 1 Pharmacy Department, Rajavithi Hospital, Bangkok 10400, Thailand; tamonwanchankaew@gmail.com; 2 Department of Pharmaceutical Care, Faculty of Pharmacy, Chiang Mai University, Chiang Mai 50200, Thailand; khimpeera_2805@hotmail.com (P.B.); dujrudee.c@cmu.ac.th (D.C.); voratima.silavanich@gmail.com (V.Y.); 3 Center of Excellence for Innovation in Analytical Science and Technology for Biodiversity-Based Economic and Society (I-ANALY-S-T_B.BES-CMU), Chiang Mai University, Chiang Mai 50200, Thailand; 4 Pharmaceutical Care Training Center (PCTC), Faculty of Pharmacy, Chiang Mai University, Chiang Mai 50200, Thailand

**Keywords:** electronic cigarettes, tobacco control, Thailand, behaviour, rationale, satisfaction

## Abstract

Electronic cigarettes (e-cigarettes) use is trending in Thailand. Electronic cigarettes are banned and illegally imported. This study aimed to investigate the behaviour, rationale, and satisfaction of e-cigarettes users and compared them between males and females. A cross-sectional study was conducted involving 1050 participants using e-cigarettes from December 2019 to February 2020. The participants were recruited by an online questionnaire posted on social media. The participants were current e-cigarettes users aged 18 years and older. Of 1050 participants, 936 were male (89.1%). The average age was 31.2 ± 8.4 years. The participants were from all regions of the country, but most (64.5%) were from central Thailand. Most e-cigarettes users comprised private employees (43.2%). The main source of e-cigarettes in Thailand is online sources such as social media. Tank-style e-cigarettes were popular among users. Amongst e-cigarettes users, the top three rationales for using e-cigarettes were fewer harmful effects from e-cigarettes than conventional cigarettes (81.0%), smoking cessation aids (80.6%), and their lack of attaching cigarette odour (58.2%). The top three reasons for satisfaction were using e-cigarettes as a conventional cigarette cessation aid (5.1 ± 1.3), lessening cravings for conventional cigarettes (5.1 ± 1.3), and reducing conventional cigarettes withdrawal symptoms (5.0 ± 1.3). Online purchase was the main source of e-cigarettes in Thailand. The general rationale for using electronic cigarettes was that they are less harmful and to quit conventional cigarettes. Thai users were satisfied to use e-cigarettes as a conventional cigarette cessation aid. Males and females differed in behaviour, rationale, and satisfaction of e-cigarettes. Public health organisations should provide accurate information about the harm of electronic cigarettes and their efficacy for tobacco cessation.

## 1. Introduction

Electronic cigarettes (e-cigarettes) are electronic nicotine delivering systems (ENDS) that generate an aerosol by heating the liquid containing nicotine, flavouring, and other additives [1]. E-cigarettes’ popularity is a cross-cultural issue; in some countries, such as United States, United Kingdom, or France, e-cigarettes are regulated by law [2]. However, a country such as Thailand, where e-cigarettes are prohibited from importation, manufacture, and sale, still faces the problem of increasing use of e-cigarettes. Although the long-term safety of e-cigarettes is not as sufficient as conventional cigarettes, several studies reported e-cigarettes were associated with lung injury and death [3,4].

Recently, e-cigarettes have become widely used amongst Thai smokers. According to a national survey of Thai e-cigarettes users in 2017, the number of smokers that used other smoking methods aside from conventional tobacco cigarettes, including e-cigarettes, had increased from 6466 to 23,337 people [5]. According to Thailand-Global Youth Tobacco Survey 2015 [6], conducted amongst 1721 students aged 13 to 15 years old, 15.0% of students were active users of any tobacco products, and 3.3% of overall students were e-cigarettes smokers. More recent articles published in Thailand about e-cigarettes use, for instance, a cross-sectional self-reported survey conducted in 2019 amongst 6167 adolescents aged 14–17 years in Bangkok, Thailand, found that adolescents had been using e-cigarettes in the last 30 days (6.7%) [7]. Similarly, a study conducted about e-cigarettes use amongst youth in Thailand showed that 7.2% had ever used e-cigarettes and 3.7% were current e-cigarettes users [8]. However, these recent articles about Thai e-cigarettes users were about adolescents.

Thai smokers have usually used conventional cigarettes, but recently e-cigarettes have become popular amongst Thai smokers. Despite Thailand’s ban on the importation, manufacture, or sale of e-cigarettes or any vaping devices, e-cigarettes users and illegal vendors are increasing, mostly through the Internet and social media [2]. This trend of using e-cigarettes is becoming prominent because of online influencers and the global use of e-cigarettes. In 2016, over 46 websites were specifically established for e-cigarettes and their components, such as liquid (E-liquid or E-juice), batteries, or even ingredients to make their own liquid [9]. Moreover, the belief that e-cigarettes are less harmful than conventional cigarettes and help quit using conventional cigarettes constituted the reasons people started using e-cigarettes [10].

Studies about e-cigarettes users’ profiles have varied from study to study [11,12,13], for example, regarding the initial age or reason for e-cigarettes use. In Thailand, few studies have been conducted to investigate the behaviour, rationale, and satisfaction of e-cigarettes users; only one study in 2014 was dedicated to studying the behaviour and satisfaction amongst 50 e-cigarettes users [14]. The number of e-cigarettes users and their profiles are limited; one study conducted in Bangkok reported 416 current e-cigarettes users (in 30 days) aged between 13 to 18 years [15], and the other was conducted amongst approximately 180 university students in northern Thailand [10]. Few studies were conducted in Thailand on e-cigarettes covering users nationally, which limits generalisability. Moreover, articles about differences between male and female e-cigarettes users in Thailand are insufficient. This study aimed to investigate and describe the behaviour of e-cigarettes use, reason of e-cigarettes use, and satisfaction level of e-cigarettes use, and compare them between males and females. This article aims to provide some perspective and additional understanding of Thai e-cigarettes users in order for the public health organization to regulate and proactively reduce e-cigarettes use in Thailand.

## 2. Materials and Methods

### 2.1. Study Design and Participants

This nationwide cross-sectional study was conducted amongst 1050 e-cigarettes users throughout Thailand. The participants were included in this study if they were aged at least 18 years, currently using e-cigarettes continuously for at least three months, were able to read, write, and understand Thai, agreed to participate in this study and had access to the Internet. The participants would be excluded from the study when they failed to provide reliable information.

The sample size was calculated to determine the number required for an unknown population size with a margin error of 3.0%, a significance level of 0.05, and a two-sided test. The sample size had to be precisely 1111 participants to provide an error margin of 3.0%; therefore, this study aimed to recruit about 1200 participants. However, the final sample size totalled 1050 participants.

### 2.2. Questionnaire Development and Data Collection

The questions and choices were developed based on a review of related literature to answer all the objectives covering the behaviour, rationale, and satisfaction of e-cigarettes users. E-cigarettes-related literature was reviewed and 20 e-cigarettes user interviews were conducted to ensure its relatability and fit to the e-cigarettes community. The content validity of the questionnaire was verified by three experts in e-cigarettes and smoking cessation. Each expert evaluated whether each question-and-answer choice provided accurate and comprehensive data relevant to the objectives based on scores ranging from −1 to +1 (+1, congruent; 0, questionable; and −1, incongruent). Final scores from these experts were used to calculate the index of item-objective congruence (IOC). Question items with scores greater than or equal to 0.5 were kept. On the contrary, question items with a score of less than 0.5 were amended in consideration of expert suggestions. After that, the questionnaire was tested amongst 20 e-cigarettes users to approve of the language and understandability of the items in the questionnaire.

The questionnaire included four sections. Section 1 included general information about the participants such as sex, age, region, occupation, and education. Section 2 included the behaviour of e-cigarettes users: initial age of using e-cigarettes, type of e-cigarettes used, source of access, and factor of buying cigarettes (quality, price, device design, or convenience to buy). Section 3 comprised the rationale of e-cigarettes use by prespecified multiple-choice items including curiosity, using e-cigarettes as a smoking cessation aid, believing e-cigarettes were less harmful than conventional cigarettes, convinced by friend or acquaintances, lacking attaching normal cigarette odour, interested in e-cigarettes’ functions and properties, and believing e-cigarettes cost less than conventional cigarettes. Section 4 consisted of satisfaction of e-cigarettes use using six Likert scale items from 1 (not satisfied), 2 (least satisfied), 3 (less satisfied), 4 (satisfied), 5 (very satisfied), to 6 (most satisfied). Eight topics of satisfaction consisted of permanently quitting smoking (for participants using e-cigarettes as a conventional cigarette cessation aid only), reducing conventional cigarette withdrawal symptoms (for participants quitting conventional cigarettes or ex-smokers only), less conventional cigarette craving, flavour or scent, device design, ease to use, e-cigarettes user image, and price.

An online questionnaire using a Google form was used to collect all required information from e-cigarettes smokers. After validating and modifying the finalized questionnaire, the link to the finalised questionnaire was posted online through famous social media platforms in Thailand such as Facebook, LINE, Twitter, and Instagram, which addressed that anyone interested in participating in the research could voluntarily answer the questionnaire. The participants would answer the question and provide their consent by selecting the statement, “I consent to participate in the study voluntarily” before entering the questionnaire and submitting the information anonymously; no personal data were required in the questionnaire and no IP addresses were required. If the participants felt uncomfortable or hesitant to respond, they had the option of refusing to provide any further information and exiting the questionnaire and the information was not registered in this study. The online questionnaire was available for three months, from December 2019 to February 2020. After three months had passed, 1050 participants had completed the questionnaire, so the researchers chose to close it and took it down from the Internet. After the information was gathered, the researcher thoroughly read and analysed all the answers to prevent repeat responses.

### 2.3. Statistical Analysis

STATA Software, Version 14.0 was used to analyse data. The results were reported using descriptive statistics: means ± standard derivations (SD) for continuous variables and frequencies and percentages for categorical data. The behaviours, rationales, and satisfaction level with e-cigarettes were compared between the sexes using the Fisher’s exact test for categorical variables or an independent *t*-test for continuous variables. The significance level was set as two-tailed and at the *p*-value < 0.05.

### 2.4. Ethics Consideration

The study protocol was approved by the Human Ethics Committee of the Faculty of Pharmacy, Chiang Mai University (Ethics approval No. 25/2019). All participants were informed about the study protocol and consented to be a part of this study.

## 3. Results

Of the 1050 participants who were e-cigarettes smokers, 846 (80.6%) were e-cigarettes only users while 204 (19.4%) were dual users, participants who used both conventional cigarettes and e-cigarettes. Most participants (89.1%) were male. The mean age of the participants was 31.2 years (SD 8.4), 43.2% were aged from 25 to 34 years, and most (64.5%) were from the central region of Thailand. Most e-cigarettes smokers (43.2%) worked at private companies, and about one half (53.8%) had obtained a bachelor’s degree or above (Table 1).

Regarding the participants’ smoking behaviour, most e-cigarettes smokers started using e-cigarettes at the age ranging from 15 to 24 (38.5%) and 25 to 34 years (39.1%); however, some smokers initiated their e-cigarettes use aged less than 15 years old. The initial age did not differ between males and females. Concerning types of e-cigarettes, most participants reported using rechargeable (66.8%) and tank-style rechargeable e-cigarettes (57.2%); more males significantly used tank-style than females. Regarding the source of accessing e-cigarettes in Thailand, the top three sources were from social media (64.6%) such as Facebook or LINE (a popular messaging application in Thailand), websites that sold only e-cigarettes (47.1%), or bought directly from the vendor (32.4%). The top three factors for buying e-cigarettes were quality (87.9%), price (64.7%), and device design (50.8%) (Table 2). The sources and factors for buying e-cigarettes did not differ between males and females.

The top three rationales why Thai e-cigarettes users used e-cigarettes were: (1) believing them less harmful than conventional cigarettes (81.0%), (2) using e-cigarettes as a smoking cessation aid (80.6%) and, (3) lacking the normal cigarette odour (58.2%) (Table 3). Among males and females, three significant differences were curiosity, using e-cigarettes as a cessation aid, and believing in less harmful e-cigarettes.

Regarding satisfaction amongst e-cigarettes users, the top three satisfaction reasons were using e-cigarettes as a conventional cigarette cessation aid (5.1 ± 1.3), lessening cravings for conventional cigarettes (5.1 ± 1.3), and reducing conventional cigarettes withdrawal symptoms (5.0 ± 1.3). Among males and females, males were significantly more satisfied with these three topics than females (Table 4). This section may be divided by subheadings. It should provide a concise and precise description of the experimental results, their interpretation, as well as the experimental conclusions that can be drawn.

## 4. Discussion

To the extent of our knowledge, this study constitutes the first nationwide research in Thailand to investigate the behaviour, rationale, and satisfaction of e-cigarettes users, and compare between males and females. Although this study focused on Thai e-cigarettes users, the inclusion criteria recruited all e-cigarettes users regardless of conventional cigarettes use. Therefore, 19.4% of participants were dual users.

### 4.1. Behaviour of E-Cigarettes Users

This study showed that some e-cigarettes users started using e-cigarettes at a young age: less than 18 years old or even less than 15 years old. This age range meant that some Thais started using e-cigarettes when they were students. Consistent with a study conducted by Ofuchi T. et al. in 2020 about e-cigarettes use amongst students aged 13 to 18 years old in Bangkok, the prevalence of e-cigarettes only users amongst adolescents was 6.7% [15]. This might be because e-cigarettes have become easier to access, e.g., users can buy them through online platforms such as websites or social media, and e-cigarettes have quickly become the most popular tobacco product among the youth, mostly due to promotion and marketing efforts by e-cigarettes businesses [16]. Thailand Tobacco Products: Policy and Control Measures for Health in 2018 reported that Thai youth aged between 15 to 18 years old easily gained access to e-cigarettes via social media [17]. Furthermore, Kinnunen JM, et al. studied 12,167 adolescents from 14 to 17 years old in Europe and showed that 34% of participants had tried e-cigarettes, and one of the associated factors of e-cigarettes smoking was low academic achievement [12]. This discovery, along with the existing study results, should be considered for any student-related organisation, educational system, and public health policymakers. Our suggestion is to create strategies to deal with adolescents using e-cigarettes by early detection and integrating lessons about e-cigarettes and cessation strategies in the national curriculum.

E-cigarettes users mostly preferred rechargeable e-cigarettes as they were the most cost-effective, easy to carry, could be used for a longer time, and be purchased online. According to findings from an online survey amongst 2807 current e-cigarettes users across many countries [18], smokers tended to use tank e-cigarettes over disposable e-cigarettes because tank e-cigarettes are more cost-effective, have a larger battery capacity than disposable e-cigarettes, and their liquid can be refilled as many times as the smokers desired. A previous study about Thai e-cigarettes users has shown a similar result. About two-thirds of Thai e-cigarettes users preferred rechargeable e-cigarettes because it is cheaper, more durable, and convenient [14]. Males significantly used tank e-cigarettes more than females. This result might be because most males used e-cigarettes to quit smoking and reduce conventional cigarette withdrawal symptoms. Therefore, tank-style e-cigarettes with more capacity and higher nicotine content might be suited for male preference. In addition, female e-cigarettes users might prefer rechargeable e-cigarettes because it is smaller.

The main source of purchasing e-cigarettes in Thailand was social media, such as Facebook and LINE, and websites that specifically sell e-cigarettes; males and females did not differ in terms of sources of e-cigarettes. Other research conducted in Thailand has demonstrated that most e-cigarettes users obtained their products and equipment from online sources [19,20]. Social media such as Facebook, LINE, and Instagram were the most popular sources for purchasing e-cigarettes or e-liquid, and websites were the second most popular source. These behaviours may be caused by the convenience of users being able to order, pay for, and receive their products easily. In addition, the law enforcement on e-cigarettes in Thailand prohibits any activity on selling e-cigarettes which means that shops, stores, or retailers would not sell e-cigarettes. Therefore, social media and the Internet became tools for users to purchase e-cigarettes and for vendors to avoid having any physical store present. Furthermore, this behaviour could result from some vendors spreading false information such as claiming the safety of e-cigarettes as a marketing tactic through advertising on their platform [19,20]. However, this result might be different from a previous study about Thai e-cigarettes usage, which stated that most e-cigarettes were bought at street vendors or markets [14]. The difference might be because of the current popularity of online shopping. Moreover, online shopping can avoid legal regulation of e-cigarettes. This concern should be considered by law enforcement in tobacco control to be aware of and plan to act against online commerce of e-cigarettes and counter any further misleading information about e-cigarettes by providing accurate facts about e-cigarettes.

Moreover, this study discovered that some smokers made their own e-cigarettes and e-liquid. They reported that making their own cigarettes and e-liquid cost less than buying factory-made e-cigarettes and claimed that hand-made e-liquid was just as effective as factory-made. This was due to the online phenomenon of e-cigarettes, online recipes to make your own e-liquid, or DIY e-cigarettes allowing smokers to create their e-cigarettes and e-liquid. Creating handmade e-cigarettes could be highly dangerous because a low-quality or unqualified device could cause many serious adverse events such as malfunctioning or battery explosion. According to “Explosion Injuries” from e-cigarettes, 25 e-cigarettes users in America reported injuries due to a lithium-ion battery blast caused by overheating batteries [4]. This could lead to burning injuries, including heat and chemical burns and explosion injuries on the teeth, face, hands, and thigh areas. Many injuries were to such severe levels that patients needed to be hospitalized and followed up for infectious complications or surgeries. The e-liquid was as unsafe as the device; the hazardous chemical found in conventional cigarettes can be found in the e-cigarettes’ aerosols [21].

### 4.2. Rationales for Using E-Cigarettes

The two rationales for using e-cigarettes amongst smokers are mostly related to conventional cigarettes: the belief that e-cigarettes are less harmful than conventional cigarettes and using them as a smoking cessation aid. This finding was inconsistent with the study by Wackowski OA, et al., reporting that most American users used e-cigarettes because of curiosity and wanted to try something new (76.5%), e-cigarettes seemed to be less harmful than conventional cigarettes (77.2%), and to reduce conventional cigarette consumption (72.2%) [22]. Furthermore, our results appeared to correlate to the results of another study from the USA with a slightly different ranking showing the majority of college e-cigarettes users chose to start using it out of curiosity, the other reasons being the enjoyment of using e-cigarettes, e-cigarettes are less toxic than conventional cigarettes, and avoiding smells like smoke [23]. Any of the differences in these findings might be due to the time differentiation, as both studies were conducted in 2017, and e-cigarettes were a new product introduced to the smoking community.

In addition, as years have passed, e-cigarettes have become popular. E-cigarettes companies created advertisements and marketing to convince consumers to believe e-cigarettes were less harmful than conventional cigarettes. Misleading information about e-cigarettes spread, for example, claims that e-cigarettes could help smokers quit conventional cigarettes, e-cigarettes were not addictive, and e-cigarettes did not cause cancer [19,20]. Hence, most e-cigarettes users in this study used e-cigarettes because they believed them to be less harmful than conventional cigarettes and could be used as a smoking cessation aid. Similar to a recent study conducted amongst 792 university students in northern Thailand [10], 143 (18.1%) respondents were current e-cigarettes users; the two most stated reasons for using e-cigarettes included: (1) being less harmful compared with conventional cigarettes and (2) being used to quit conventional cigarettes. The recently established evidence about the potential harm of e-cigarettes was nicotine dependence, toxicants and carcinogens from vapour, burn, and lung injuries [24]. There had been incidents of e-cigarettes related injuries such as an e-cigarettes or vaping use-associated lung injury (EVALI) in which e-cigarettes users experienced symptoms such as coughing, chest pain, breathing difficulty, stomach ache, nausea, vomiting, and in the worst cases, death. The danger of the e-cigarettes related to EVALI is the e-liquid or e-juice that might contain substances that can be lethal when vaporized [5]. This issue should be properly informed to the public and public health organisations must act toward the community, providing accurate, updated, and unbiased information about the harm of e-cigarettes.

When comparing between the two sexes, few similarities and differences in the topic of rationale were discovered. The top three rationales for using an e-cigarette amongst both males and females were: (1) believing e-cigarettes were less harmful than conventional cigarettes, (2) using them as a smoking cessation aid, and (3) lacking a normal cigarette odour. These findings implied that both males and females were concerned about their health status and fully aware of the harm of conventional cigarettes. However, some significant differences were found. Females mostly used e-cigarettes out of curiosity more than males. Female Thai e-cigarettes users might use e-cigarettes out of curiosity significantly more than males because males had tried e-cigarettes more than females. Our result differed from a prior study [25], reporting that the rationale for using e-cigarettes amongst males was the belief that e-cigarettes can be used to help quit conventional cigarettes, had more health benefits, and were more curious than females. This might be because, in our study, females started using e-cigarettes at a younger age, so they might use e-cigarettes because of curiosity. Whilst amongst females, the reasons for using e-cigarettes were recommended by friends and family members. Similarly, Thai females were more likely to ask for conventional cigarettes from friends [26]. As reported in a related study [27], a significant association was found between e-cigarettes use and sex, with males having used e-cigarettes more than females.

### 4.3. Satisfaction

Of 1050 smokers in this study, 842 (80.2%) participants used e-cigarettes as a smoking cessation aid and 74.8% were very satisfied with the result that e-cigarettes could help quit smoking conventional cigarettes. In line with a study including 28 European countries with 27,460 participants aiming to assess the prevalence of e-cigarettes use and changes in smoking status because of e-cigarettes, the finding showed that e-cigarettes users were satisfied that it could reduce their conventional cigarette cravings and they could quit smoking eventually [28]. Similarly, a study on e-cigarettes using websites and online discussion forums for users to discuss e-cigarettes and smoking cessation, revealed over 92.2% of e-cigarettes smokers were satisfied with using e-cigarettes as a smoking cessation aid [11]. These findings demonstrated that even though time has passed, users were still using another form of smoking to quit conventional cigarettes rather than seeking help in appropriate smoking cessation methods proven to be effective and safe. This might be due to a lack of public relations campaigns or advocate programmes on effective smoking cessation methods for smokers who want to quit smoking.

Few high-quality studies indicated the efficacy of using e-cigarettes as a conventional cigarette cessation aid for even heavy e-cigarettes users [11,29,30]. Nevertheless, some studies might have shown that e-cigarettes could minimize withdrawal symptoms from conventional smoking cessation [11]. Some studies have demonstrated that e-cigarettes could help smokers quit conventional cigarettes, but they still smoked e-cigarettes to substitute for conventional cigarettes. However, satisfaction was assessed using self-evaluation-based questionnaires on participants’ feelings and experiences, so participants’ bias may have occurred. Furthermore, regarding satisfaction of using e-cigarettes as a conventional smoking cessation aid, to date, no evidence has established that e-cigarettes users, who were very satisfied with the result that e-cigarettes would help quit smoking conventional cigarettes, could quit smoking conventional cigarettes.

The study on the topic of satisfaction between males and females remains limited. Male users were satisfied with e-cigarettes being a smoking cessation aid; this could be because males prefer to use tank-style e-cigarettes because of the high capacity and nicotine content and suitability as a smoking cessation aid. This study showed that male e-cigarettes users were more satisfied with e-cigarettes on the topic of permanent cessation of smoking, less conventional cigarette craving, and reducing conventional cigarettes’ withdrawal symptoms.

### 4.4. Strengths and Limitations of This Study

To the best of our knowledge, this study is among the few studies dedicated to investigating the behaviour, rationale, and satisfaction of e-cigarettes users in Thailand nationally with responses from participants. Because online questionnaires and social media were used as data collecting tools in this study, it covered all regions of Thailand and almost every age group (≥18 years old) of Thai e-cigarettes users.

However, this study encountered some limitations. First, to participate in the online questionnaire, participants must gain access to the internet; thus, some e-cigarettes users who did not have access to the internet were excluded from this study. Second, a self-report questionnaire may have caused response bias due to participants’ perspectives and recalled experiences. However, the participants responded voluntarily to the online questionnaires, making this limitation less likely to have occurred; to measure smoking consumption, self-reporting is a valid method [31,32]. Third, although this study was able to include many participants and cover all regions of Thailand due to the use of an online questionnaire to collect all relevant information, the representativeness of Thai e-cigarettes users should be of concern because most of the participants were from central Thailand.

## 5. Conclusions

Thailand e-cigarettes users mostly purchased online. The main reasons for using e-cigarettes were the belief they were less harmful and to quit conventional cigarettes. Users were satisfied to smoke e-cigarettes as a conventional cigarette cessation aid. Both sexes differed in behaviour, rationale, and satisfaction of e-cigarettes. Tank-style e-cigarettes were preferred among male users. The reasons for e-cigarettes use in Thailand were different between males and females; females used e-cigarettes out of curiosity, while males used e-cigarettes for a smoking cessation aid and believed that it was less harmful than the conventional cigarette. Males were more satisfied using e-cigarettes in terms of less conventional cigarette craving, permanently quitting smoking, and reducing conventional cigarette withdrawal symptoms.

At the time, studies showed that younger smokers still believed that e-cigarettes were less harmful than conventional cigarettes, despite the updated information about the danger of e-cigarettes. Moreover, whether e-cigarettes can be used as a smoking cessation aid remains controversial. Therefore, e-cigarettes cessation strategies and law enforcement should target the misbeliefs and misperceptions of e-cigarettes amongst both males and females.

## Figures and Tables

**Table 1:** Characteristics of e-cigarettes users (*n* = 1050).

Characteristic	Total (*n*)	Percentage
Smoking status		
E-cigarettes only users	846	80.6
Dual users	204	19.4
Sex		
Male	936	89.1
Female	114	10.9
Mean age ± SD (years)	31.2 ± 8.4	
Age (years)		
18–24	252	24.0
25–34	454	43.2
35–44	267	25.4
45–54	64	6.1
≥55	13	1.2
Region		
Central	677	64.5
East	114	10.9
North	96	9.1
Northeast	79	7.5
South	66	6.3
West	18	1.7
Occupation		
Private company	454	43.2
Businesses	243	23.1
Student	156	14.9
Government officials	102	9.7
Others *	95	9.1
Education		
Bachelor’s degree or above	565	53.8
Senior year high school or vocational certificate	262	25.0
Diploma or high vocational certificate	145	13.8
Junior high school or below	78	7.4

* Other occupations included agriculturist, homemaker/husband, athlete, architect, engineer, musician, teacher and unemployed.

**Table 2:** Behaviour of e-cigarettes users by sex (*n* = 1050).

Behaviour	Total (*n* = 1050)	Female (*n* = 114)	Male (*n* = 936)	*p*-Value
Initial age of e-cigarettes use (years)				
<15	23 (2.2)	3 (2.6)	20 (2.1)	0.086
15–24	404 (38.5)	54 (47.4)	350 (37.4)	
25–34	411 (39.1)	45 (39.5)	366 (39.1)	
35–44	174 (16.6)	10 (8.8)	164 (17.5)	
45–54	37 (3.5)	2 (1.8)	35 (3.7)	
≥55	1 (0.1)	0 (0.9)	1 (0.1)	
Type of e-cigarettes				
Rechargeable e-cigarettes	701 (66.8)	80 (70.2)	621 (66.4)	0.462
Tank-style rechargeable e-cigarettes	601 (57.2)	50 (43.9)	551 (58.9)	0.003 ^†^
Pen-style rechargeable e-cigarettes	272 (25.9)	37 (32.5)	235 (25.1)	0.112
Disposable e-cigarettes	59 (5.6)	8 (7.0)	51 (5.5)	0.516
Source of access				
Social media, e.g., Facebook or LINE	678 (64.6)	72 (63.2)	606 (64.7)	0.756
Website that sells only e-cigarettes	494 (47.1)	50 (43.9)	444 (47.4)	0.488
Bought directly from vendors	340 (32.4)	35 (30.7)	305 (32.6)	0.751
Imported (Pre-ordered)	262 (25.0)	30 (26.3)	232 (24.8)	0.731
Vendors	172 (16.4)	15 (13.2)	157 (16.8)	0.421
The factor for buying e-cigarettes				
Quality	923 (87.9)	96 (84.2)	827 (88.4)	0.222
Price	679 (64.7)	73 (64.0)	606 (64.7)	0.917
Device design	533 (50.8)	64 (56.1)	469 (50.1)	0.235
Convenience to buy	381 (36.3)	50 (43.9)	331 (35.4)	0.080

^†^ Indicate the statistical significance level a *p*-value < 0.05.

**Table 3:** Rationale of e-cigarettes users compared by sex (*n* = 1050).

Rationale *	Total (*n* = 1050)	Female (*n* = 114)	Male (*n* = 936)	*p*-Value
1. Believing e-cigarettes were less harmful than conventional cigarettes	850 (81.0)	77 (67.5)	773 (82.6)	<0.001 ^†^
2. Using as a smoking cessation aid	846 (80.6)	78 (68.4)	768 (82.1)	0.001 ^†^
3. Lacking attaching normal cigarette odour	611 (58.2)	69 (60.5)	542 (57.9)	0.616
4. Interested in e-cigarette’s function and properties	544 (51.8)	67 (58.8)	477 (50.9)	0.136
5. Believing e-cigarettes cost less than conventional cigarettes	542 (51.6)	51 (44.7)	491 (52.5)	0.136
6. Convinced by a friend or acquaintance	179 (17.0)	27 (23.7)	152 (16.2)	0.064
7. Curiosity	154 (14.7)	26 (22.8)	128 (13.7)	0.016 ^†^

* Each participant could select more than one answer. ^†^ Indicate the statistical significance level a *p*-value < 0.05.

**Table 4:** Satisfaction of e-cigarettes users by sex *.

Satisfaction	*n*	Total (*n* = 1050)	Female (*n* = 114)	Male (*n* = 936)	*p*-Value
1. Less conventional cigarette craving	1041	5.1 ± 1.3	4.6 ± 1.6	5.2 ± 1.3	<0.001 ^†^
2. Permanently quit smoking **	842	5.1 ± 1.3	4.5 ± 1.6	5.1 ± 1.3	<0.001 ^†^
3. Reduce conventional cigarette withdrawal symptoms ***	1026	5.0 ± 1.3	4.6 ± 1.5	5.1 ± 1.3	<0.001 ^†^
4. Flavour or scent	1048	5.0 ± 1.1	4.9 ± 1.2	5.0 ± 1.1	0.432
5. Device design	1049	4.8 ± 1.1	4.7 ± 1.4	4.8 ± 1.1	0.236
6. Easy to use	1050	4.5 ± 1.5	4.6 ± 1.4	4.5 ± 1.5	0.569
7. E-cigarettes user image	1049	4.3 ± 1.4	4.3 ± 1.5	4.3 ± 1.4	0.913
8. Price	1048	4.3 ± 1.3	4.3 ± 1.4	4.3 ± 1.3	0.709

* Each participant could select more than one answer. ** For participants using e-cigarettes as a conventional cigarette cessation aid only. *** For participants quitting conventional cigarettes or ex-smokers only. Likert Scale from 1 (not satisfied) to 6 (most satisfied). ^†^ Indicate the statistical significance level a *p*-value < 0.05.

## Data Availability

The data presented in this study are available from the corresponding author on reasonable request.
